# Impact of smart device use on objective and subjective health of older adults: findings from four provinces in China

**DOI:** 10.3389/fpubh.2023.1118207

**Published:** 2023-07-20

**Authors:** Yan Wei, Xinyu Guo

**Affiliations:** School of Statistics, Xi'an University of Finance and Economics, Xi'an, China

**Keywords:** smart device, older adults, self-rated health, physical health, psychological health, propensity score matching, mediating effect

## Abstract

**Background:**

The digital divide has grown because of the deepening digitalization of the Chinese society and the intersection between high-end technology and the age structure. Older adults show an increasing level of weakness in digital life integration. What digital development can bring to older adults is a pressing concern.

**Objective:**

This study aims to investigate how smart device use affects older adults' health status and offers an empirical reference for improving their digital literacy and health.

**Methods:**

The data in this study were collected from an offline survey conducted from December 2021 to April 2022, which obtained 1110 valid samples of older adults This study used a multivariate ordered logit model, mediating effect model, and heterogeneity test to analyze the impact of smart device use on the health status of older adults.

**Results:**

Smart device use has a significant positive effect on the self-rated, physical, and psychological health of older adults, and this positive effect is more pronounced among older adults living in urban areas or with a higher age. The average net effect of smart devices on each health status of older adults is 0.161 for self-rated health, 0.155 for physical health, and 0.071 for psychological health. In-depth research found that older adults' attitudes toward smart devices played a mediating role in the influence of smart device use on self-rated and psychological health respectively.

**Conclusions:**

The study found that smart device use had a positive effect on the health status of older adults and that the performance expectations and individualized needs of older adults exhibited an effective linkage between smart devices and health status. Smart device use could improve the overall health of older adults, especially the urban and low-age older adults. Promoting the understanding of the practicality of smart devices for older adults and the aging-oriented transformation of smart devices is an effective way to improve the health status of older adults. The findings provide theoretical support for the wide application of smart devices in older adults, and can effectively help eHealth practitioners implement accurate geriatric health support strategies.

## 1. Introduction

The intersection of high-end technology and the age structure widened the age-oriented digital divide. The large population has made the digital divide among older adults a major livelihood concern for governments in the digital age. Older adults lag behind younger people in their adoption, especially when it comes to internet use (1). Also, older adults have been classified as one of the most information-weak groups in some countries including Korea (2) and China. Exploring the impact of digital development on older adults will help integrate existing resources for senior care and significantly enhance the support for older adults' health and digital literacy.

The United Nations projects that the world's population could grow to around 8.5 billion by 2030 (3), up from the recent number of 8 billion. Average life expectancy also increases with population growth. It is predicted that the average longevity worldwide will be 77.2 years by 2050 (4). Population aging will increase because of increased life expectancy and declining birth rate. The Second World Assembly on Aging took place in Madrid in 2002, where it was proposed that “population aging is a universal force that has the power to shape the future as much as globalization.” (5) As the world's most populous region, the overall speed and level of population aging in Asia have a huge impact on the quantity and quality of life of the global population. In China, the population of older adults aged 60 or above had reached 2.67 billion by the end of 2021(6), making China the country with the largest number of older adults in the world. Aging in China shows obvious characteristics of getting old before becoming rich and processing faster. China put forward a policy of actively coping with population aging in 2006, which was elevated to a national level in the 14th Five-Year Plan (2021–2025) in 2021.

Currently, the main problems that need to be resolved are how to better satisfy the needs of older adults, encourage their engagement, and help them share the benefits of social development considering China's rapid aging. This study focused on the impact of smart device use on the health status of older adults. The rationale behind choosing this research theme is that older adults exhibit growing digital fragility and obvious digital weakness as the digitalization of Chinese society deepens. Smart devices are among the most direct links between the internet and its users. Research on the impact of smart devices on the health status of older adults will help clarify the impact of digital life on the health of older adults, help explore the aging-oriented development of smart products, and effectively help solve the digital divide for older adults.

The possible innovations and contributions of this study are as follows: first, in the context that most of the current research is on a single health aspect, the study for the first time comprehensively considers the subjective and objective health of older adults and analyzes the impact on each health status by combining multidimensional perspectives of social interaction, economic level, and intergenerational support. Second, in the context of a deeply aging society and accelerating the construction of digital China, this paper further analyzes the intrinsic effect mechanism combined with actual life needs, and analyzes the essence in depth based on the analysis of the impact of smartphone use on health to provide empirical references for the improvement of health quality and digital literacy of older adults. Finally, the findings show that use smartphone in moderation can improve the overall health of older adults, and provide a feasible development direction for improving the digital literacy of older adults and achieving healthy aging.

This study is divided into four parts. First, the literature review section gives the theoretical origins of the concept of “eHealth” and reviews the literature on smart device use and health status of older adults. Second, materials and methods are included, which consist of data sources, variable definitions, and analysis strategies. Third, the impact of smart device use on the health status of older adults were analyzed, in which robustness tests and mediating effects tests were conducted, and heterogeneity tests based on older characteristics were analyzed. Finally, this paper discusses the analyses and summarizes the limitations.

## 2. Literature review

Palos-Sanchez's study (7) noted that telemedicine and eHealth have duly become important factors for the analysis, study, etc.,. “Health services and information provided by the Internet and related technologies.” was used by Eysenbach (8) to define Electronic health or eHealth. It can be seen that eHealth not only refers to the development of medical information technology, but also means an Internet-based way, which is more conducive to promoting the development of modern health care (9). As one of the main accesses for older adults to the Internet, smart devices offer older adults a new way to access health information and improve their health conditions.

The relationship between smart devices and the health status of older adults is an important topic in digitalization and aging. Studies have been conducted to analyze the overall health of older adults in terms of smart devices, individual characteristics, and social status. The popularity of smart mobile devices such as smartphones and smartwatches has promoted the openness and expansion of mobile health applications, providing support for coping with structural changes and population aging (32). The popularity of mobile technology has led to the widespread development of digital health interventions (10), and the use of smart wearable devices can help older adults improve their health (11). In terms of social interaction, active participation in social activities contributes to healthy aging. The social networks of older adults have different influences on their health. The use of digital technologies such as the internet can effectively contribute to active aging, and studies have shown that older adults who use online payments have better physical and psychological health (12). Older adults who use the internet also have a larger social network in which their social participation plays a positive role (13), while living alone has a negative impact on their health and overall wellbeing (14). Education plays a role in the choice of social networks and the number of choices made by older adults (14). Moreover, some studies have shown that internet health information education for older adults can significantly improve their health literacy (15). The overall health of older adults has improved after using health self-tests and health training provided by smartphones (16).

The health of older adults can be divided into self-rated, physical, and psychological health. First, in terms of self-rated health, some studies have shown that reported symptoms and disease factors, biological factors, and behavioral factors can lead to gender differences in the subjective health of older adults and that the health quality of the female population is lower than that of the male population when controlling for residential characteristics, basic physical health, socioeconomic status, and family support. Smartphone use among older adults has a significant positive effect, which increases with the improvement in education, income, and children's living conditions on their subjective health status determination (17). Some studies have found that internet access among older adults can improve self-rated health and has multiple positive effects on health and quality of life (18). A study by Nwoke et al. (19) pointed out that education was the least negligible factor when predicting the impact of the internet on the self-rated health of older adults, and the higher the education level, the better the psychological health status. Yang and He (20) found that internet use could significantly improve self-rated health and mood in adults aged 45 years or above.

Second, in terms of physical health, the development of the internet has helped older adults obtain health information. Moderate smartphone use and its support software are conducive to improving the health of older adults. Some studies have shown that internet use can effectively improve the physical health of older adults (21). The development of the internet has also facilitated access to health information, prevention of illnesses, and helped residents receive medical services more conveniently (20). Cohall et al. (22) believed that, through the internet, older adults could obtain more knowledge that benefits their physical health. However, Billari et al. (23) argued that long-term internet use could have a negative impact on physical health. Smartphones and their support software can also help older adults improve their health, with 12 smartphone applications being found to help older adults improve their health and quality of life while maintaining safe social distancing during the COVID-19 pandemic (24).

In terms of psychological health, intergenerational support and social interaction have a significant impact; moderate use of the internet and smartphones can also help older adults enhance socialization and alleviate loneliness. Appropriate online socialization can improve the psychological health of older adults. Studies have shown that once older adults establish a social network on the Internet, they are bound to weaken their psychological loneliness, relieve pressure and depression, and their psychological health benefits from it (25). The positive impact of the internet on the psychological health of older adults also increases over time (26). Sharing information through social media can consolidate family relations and alleviate the sense of social alienation and loneliness of older adults, improve self-efficacy and the sense of control, and ultimately enhance their life satisfaction (27). In terms of smartphone use, intergenerational technical support can significantly affect the feelings of older adults (28). Smartphones improve the emotional health of older adults more than VR technology (29); however, excessive smartphone use can also be harmful, with older adults who are addicted to smartphones being more likely to suffer from severe anxiety and depression symptoms (30).

Throughout the relevant studies, the overall health status of older adults was mostly analyzed from the aspects of individual characteristics, social interactions, and the use of internet and smart devices. We have summarized some of the key literature (Table 1). Some studies were conducted separately on self-rated, physical, and psychological health and concluded that older adults could effectively improve their health conditions by moderate contact and use of the internet and smart devices. At the same time, individual characteristics, social interaction, and intergenerational support had mostly significant effects on the health of older adults. Studies on the impact of smart device use on older adults' health status still have the following research spaces: first, most of the studies focus on a certain aspect of health status, and there is a lack of studies that analyze the impact of smart device use represented by smartphones on the comprehensive health status of older adults. Second, although some researchers have explored the impact of smart device use on the health status of older adults using both qualitative and quantitative methods, the underlying mechanism is relatively complex and the existence of self-selection bias remains to be further examined. Third, few studies have further explored whether other factors affect the relationship between smart device use and the health status of older adults. Therefore, this paper will examine the impact of smart device use on self-rated, physical, and psychological health to enrich the study on the impact of smart devices on health status of older adults and test the mediating effects of the performance expectations and individualized needs of older adults from smart devices. More conclusions were obtained from the group heterogeneity tests for comprehensive health according to age and residence.

**Table 1:** Summary of key literatures.

**Item**	**Overall health**	**Self-rated health**	**Physical health**	**Psychological health**
Smart device	Lead to widespread development of digital health interventions; online health training improves overall health; internet access positively affects health and life quality	Internet access improves self-rated health; internet use could improve self-rated health and mood	Help obtain health information and receive medical services; long-term internet use affects negatively	Positive effect increases over time; older adults addicted to smartphones being more likely to suffer from severe anxiety and depression.
Social interaction	Active participation contributes to active aging			Weaken psychological loneliness, relieve pressure and depression; Appropriate online socialization improves health
Education	Affect the choice of social networks and the number of choices	Education was an essential factor when predicting the impact of the internet		
Living arrangement	Living alone has a negative effect on health			Appropriate intergenerational technical and communication can positively affect older adults' feelings

## 3. Materials and methods

### 3.1. Aims and questions

This study aims to investigate how smart device use affects older adults' health status and offers an empirical reference for improving their digital literacy and health.

Specifically, this study wants to examine the following questions: whether the use of smart devices affects the health status of older adults? If so, are there any differences between older adults with different characteristics? If there are other factors that play a role in the mechanism between smart device use and the health of older adults?

### 3.2. Data

**Figure 1:**
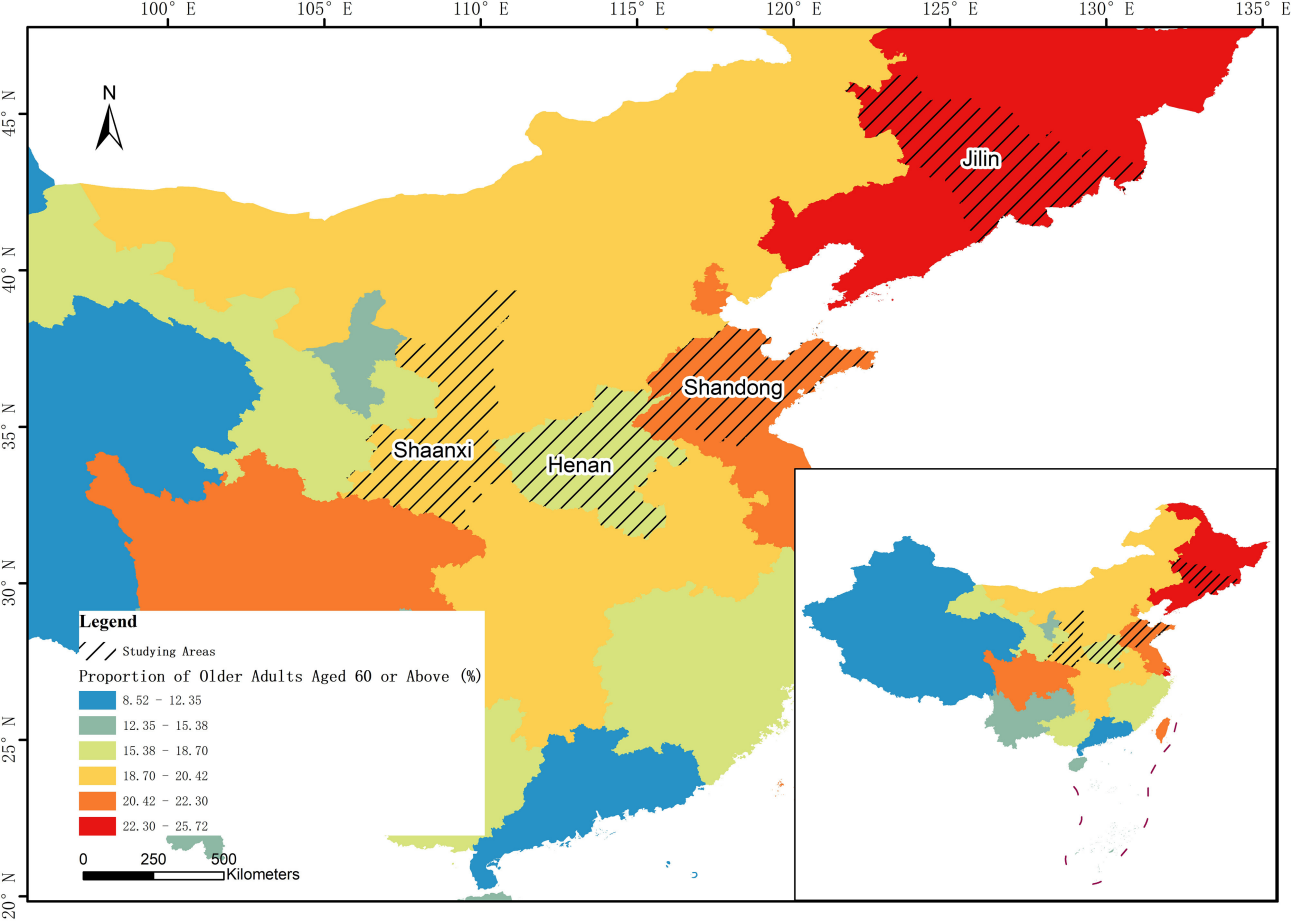
The distribution of survey locations.

The data used in this study were obtained from a face-to-face offline survey of older adults in four provinces of China from December 2021 to April 2022.This project convened and trained investigators offline to obtain rigorous and authentic questionnaires through face-to-face interviews with older adults. Before the formal survey, a small-scale pre-survey was conducted to check the validity of the questionnaire. Investigators used the initial questionnaire to carry out a small-scale offline survey of the target older adults with different gender, age, residence and other characteristics. Then some questions and options were adjusted according to the survey results, which improved the rationality and rigor of the questionnaire. Based on the geographical distribution and aging degree in the results of Seventh National Population Census, four provinces were selected as survey locations: Jilin Province in the Northeast, Shandong Province in the East, Henan Province in the central, and Shaanxi Province in the West. According to factors such as the proportion of population in each province and the needed number of respondents, the number of respondents in each province is calculated, and then subdivided into municipalities within the provinces. The aging degree of Henan Province is lower than the average level of China (18.70%), while that of other three provinces is higher, which represents the different aging degrees in different regions of China. Figure 1 shows the distribution of survey locations; the colors of provinces with a higher aging degree are darker.

The survey, targeting people aged 55 and over, included both questionnaires and qualitative interviews. Questionnaires consist of individual and community questionnaires, and the former of which can be further divided into three parts: basic information about older adults and their family situation, smart device use in older adults, and basic information about their offspring. A total of 1,170 responses were obtained, including 1,110 valid responses used in this study. The sample statistics are shown in Table 2. It can be seen from the table that there were 522 male older adults, accounting for 47.02%, and 588 female older adults, accounting for 52.97%, indicating that the gender distribution of the survey respondents was relatively even. In terms of age, there were more older adults with a higher age. There are 191 older adults aged 55–64, accounting for only 17.21%, 424 older adults aged 65–74, accounting for 38.20%, and 495 older adults aged 75 and above, accounting for 44.59%, which was much in line with the aging trend of China's older adults. As for education level, the vast majority of older adults had a low education level, 593 older adults only had primary education or below, accounting for 53.42%, only 66 older adults have college education or above, accounting for 5.95%; In terms of marital status, 793 older adults had spouses, accounting for 71.44%, and 317 older adults were without spouses, accounting for a relatively low proportion of 28.56%; As for average monthly income, 59.37% of the older adults had a monthly income of < 2,000 yuan, 28.65% of the older adults had 2,000–3,999 yuan, and only 11.98% of the older adults had more than 4,000 yuan, which mean that most of the older adults had a low monthly income.

**Table 2:** Sample description.

**Item**	**Content**	**Size**	**Proportion (%)**
Gender	Male	522	47.02
	Female	588	52.97
Age	55–64	191	17.21
	65–74	424	38.20
	75 or above	495	44.59
Education level	Primary school or below	593	53.42
	Junior high school	302	27.21
	High school or technical secondary school	149	13.42
	Junior college or above	66	5.95
Marital status	Has spouse	793	71.44
	Without spouse	317	28.56
Average monthly income	0–1,999	659	59.37
	2,000–3,999	318	28.65
	4,000 or above	133	11.98

Compared with the data of Seventh National Population Census and the 49^th^ Statistical Report on China's Internet development released by the China Internet Network Information Center (CNNIC) (31) (Table 3), the characteristics of the survey respondents are relatively similar and the data have good representative traits.

**Table 3:** Comparison of the survey with the results of Seventh National Population Census and the 49th statistics on China's Internet development (%).

**Item**	**Content**		**Survey result**	**Seventh National Population Census**
Health status	Good health		54.4	54.6
	General health		28.5	32.6
	Poor health		17.1	12.8
Education level	Primary school and below		53.5	58.6
	Junior high school		27.2	27.5
	High school or technical secondary school		13.4	9.92
	Junior college or above		6.00	3.98
**Item**	**Content**		**Survey result**	**CNNIC**
Internet Use	Internet penetration rate for older adults		47.1	43.2
		Instant messaging	95.7	90.6
	Commonly used applications	Web video	83.2	84.8
	of older Internet users	Web news	62.4	77.9
		Online payments	54.6	70.6

### 3.3. Variables

Most existing studies on the health status of older adults have been conducted in terms of self-rated, physical, and psychological health. This study described the health status of older adults in terms of three dependent variables for a comprehensive analysis of the same sample. The dependent variables in this study were the health status of older adults, including their self-rated, physical, and psychological health (Table 4).

**Table 4:** Variable definition and descriptive statistics.

**Variable**		**Variable definition**	**Mean**	**SD**
Dependent variable	Self-rated health	Poor = 1, Average = 2, Good = 3	2.377	0.760
	Physical health	Poor = 1, Average = 2, Good = 3	2.613	0.571
	Psychological health	Poor = 1, Average = 2, Good = 3	1.862	0.441
Independent variable	Smartphone use	No = 0, Yes = 1	0.570	0.495
Mediator	Performance expectations	Identification of getting valuable information from smart devices	2.294	0.768
	Individualized needs	Identification of smart devices meeting life needs	2.224	0.810
Control variable	Gender	Female = 0, Male = 1	0.470	0.497
	Age	55–64 = 1, 65–74 = 2, 75 or above = 3	2.274	0.737
	Residential area	Rural = 0, Urban = 1	0.445	0.497
	Education level	Primary school or below = 1, Junior high school = 2, High school or technical secondary school = 3, Junior college or above = 4	1.719	0.910
	Marital Status	Without spouse = 0, Has spouse = 1	0.714	0.452
	Average monthly income	0–1,999 = 1, 2,000–3,999 = 2, 4,000 or above = 3	1.526	0.700
	Interpersonal expenditures	0–999 = 1, 1,000–5,999 = 2, 6,000 or above = 3	1.969	0.676
	Living arrangement	With families = 1, Alone = 2, Others = 3	1.176	0.432

SD, Standard deviation.

The first dependent variable was the self-rated health status of older adults. Specifically, the responses to the question “How is your current physical health?” was used to evaluate the self-rated health. The five categories that respondents used for their answers were combined into “Poor” (a combination of “Very poor” and “Poor”), “Average” and “Good” (a combination of “Very good” and “Good”), considering the small number of people who chose “Very poor” or “Very good.”

The second dependent variable was the objective health degree of older adults. Physical health was measured using 12 indicators on the Activities of Daily Living (ADL) scale: eating, bathing, dressing, going to the toilet, walking, going up and down stairs, traveling by car, shopping, doing housework, washing, cooking, and talking on the phone. The value of each option was “1 = Fully able,” “2 = Partially able,” and “3 = Unable.” The reliability (Cronabach's α = 0.928) and validity (KMO = 0.922) analysis of this scale showed good results. The total score of the 12 items was 12 for “Good” coded as 3, 13–24 for “Average” coded as 2, and 25–36 for “Poor” coded as 1.

The third dependent variable was the psychological health. This variable, was based on five indicators of the depression scale in the questionnaire: mood, life emptiness, loneliness, sleep status, and life attitude. Two of the questions were rated positively and three were rated negatively. The values assigned to each option were “1 = Strongly disagree,” “2 = Disagree,” “3 = Average,” “4 = Agree,” and “5 = Strongly agree.” The positive scoring questions were turned, the reliability (Cronabach's α = 0.708) and validity (KMO = 0.661) analysis of this scale showed good results. Then the values of the five questions were combined. The total score of 16–25 for “Poor” coded as 1, 6–15 for “Average” coded as 2, and 5 for “Good” coded as 3.

The independent variable in this study was smart device use by respondents, represented by smartphones, as measured by the question “Do you use a smartphone” in the questionnaire with the options including “Yes” and “No.”

Additionally, a list of factors considered important in affecting the health status of older adults was used as a control variable in this study. The personal characteristics of the older adults included gender (male, female), age (55–64, 65–74, 75 or above), resident area (rural, urban) (20), education (literacy) (17, 19), marital status (has spouse, without spouse) (12), and other variables reflected in the economic level (personal average monthly income) (12), social interactions (interpersonal expenditure) (25, 27), and intergenerational support (living arrangement) (14). It was tested that there was no muti-collinearity between the independent variable and the control variables.

According to existing studies, smartphone use by older adults could improve their quality of life (24), and the health of older adults with online social networks is better (25). In addition, health information obtained through the internet (22) or smartphones (16) helps older adults improve their health. Combined with Maslow's hierarchy of needs, cognitive (high-level), physiological, and social (low-level) needs are closer to the real needs of users in terms of social information and individual satisfaction. This study selected the performance expectations and individualized needs of older adults as mediators, considering the results of this survey that more than 50% of older adults use smartphones to fulfill the following needs: receiving phone calls, chatting on WeChat, online payment, entertainment, and getting news and information. Performance expectations were expressed by “How much do you agree that you can get valuable information by using smart devices?” and individualized needs were expressed by “How much do you agree that using smart devices can meet your needs in life?” The values assigned to each option were “1 = Disagree,” “2 = Average,” and “3 = Agree.”

### 3.4. Analytic strategy

This study first conducted a descriptive statistical analysis of the health status of older adults under different smartphone use conditions. Second, a multivariate ordered logit model was used to examine the impact of smart device use on the health status of older adults owing to the ordinal characteristics of its measurement, The reason for using the ordered multivariate ordered logit model here is that it can be used as a generalized form of logit model to analyze in the situation that the dependent variable is a multivariate ordered variable, and the dependent variables in this paper are all categorical variables and have more than two classifications, and also each classification is ordered. A propensity value matching model was used to analyze the net effect under control variables. Among the control variables, we also introduced interpersonal expenditure, average monthly income, and residential area of older adults in addition to the basic characteristics of older adults to comprehensively explore the impact of smart device use on the health status of older adults in a multi-dimensional way. Third, we analyzed the mediating effect of performance expectations and individualized needs between smart device use and the health status of older adults to further explore the mechanism. Finally, we examined age and residence heterogeneity in the impact of smart device use on the health status of older adults.

## 4. Results

### 4.1. Descriptive analyses of health status of older adults under different smartphone use conditions

**Figure 2:**
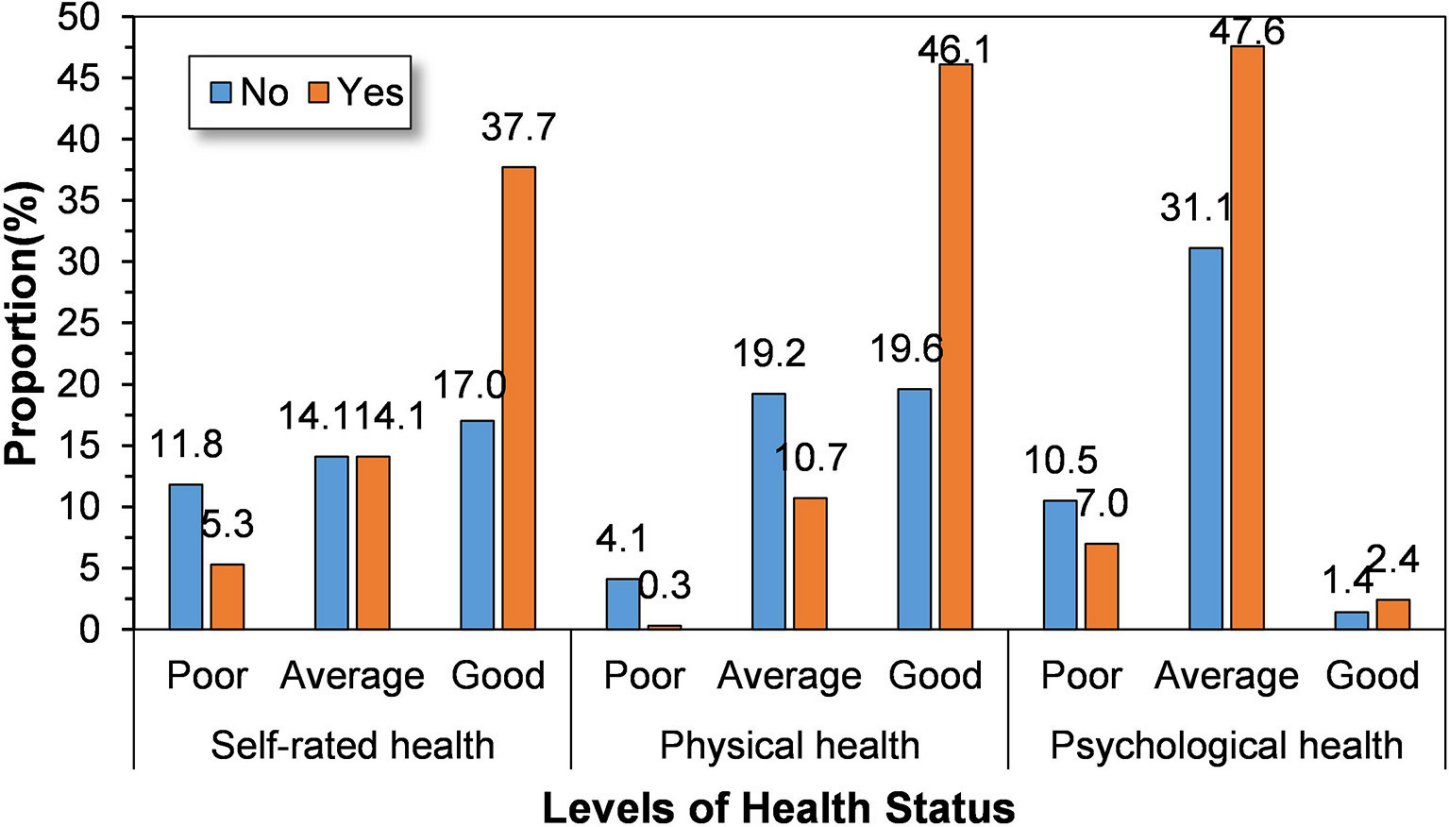
The health status of older adults under different smartphone use conditions.

Of the 1,110 respondents, 633 (57.0%) used smartphones, while 477 (43.0%) did not. As shown in Figure 2, the proportions of older adults using smartphones who reported good self-rated, physical, and psychological health were 37.7%, 46.1%, and 2.4%, respectively, while the corresponding proportions among those who did not use smartphones were 17.0%, 19.6%, and 1.4%, respectively. By comparing older adults' health under different smartphone use conditions, it was found that older adults who use smartphones account for a greater proportion of older adults displaying better health, which indicates that there is a positive correlation between smartphone use and the health condition of older adults.

### 4.2. Baseline regressions of the impact of smartphone use on the health status of older adults

In this study, six baseline regression models were built to analyze the impact of smartphone use on each health condition of older adults under control variables. Table 5 reports the impact of smartphone use on the health of older adults. Models (1) and (2) show the regression results for older adults' self-rated health. After adding the characteristics of economic level, social interaction, and intergenerational support of older adults, the estimated coefficients of smartphone use were still significant, and the interpretability of the model was enhanced. The significance of each control variable in Model (2) is consistent with that in Model (1), and the models show good stability. Models (3) and (4) are the regression results for older adults' physical health, and Models (5) and (6) are the regression results for older adults' psychological health, both of which can be interpreted similarly.

**Table 5:** Regression analyses of smartphone use on the health status of older adults (*N* = 1,110).

**Variable**	**Self-rated health**		**Physical health**		**Psychological health**	
	**(1)**	**(2)**	**(3)**	**(4)**	**(5)**	**(6)**
Smartphone use (no)	0.727^***^	0.624^***^	1.044^***^	0.952^***^	0.380^**^	0.317^*^
	(0.136)	(0.140)	(0.155)	(0.159)	(0.176)	(0.179)
Male (female)	0.023	−0.043	−0.173	−0.226	−0.073	−0.086
	(0.124)	(0.127)	(0.145)	(0.148)	(0.154)	(0.157)
**Age (55–64)**						
65–74	0.004	0.104	−0.773^**^	−0.737^**^	−0.025	0.047
	(0.193)	(0.197)	(0.303)	(0.307)	(0.236)	(0.239)
75 or above	−0.536^***^	−0.467^**^	−1.907^***^	−1.881^***^	0.015	0.078
	(0.199)	(0.202)	(0.299)	(0.302)	(0.248)	(0.250)
Urban (rural)	0.172	0.051	−0.063	−0.189	0.074	0.015
	(0.125)	(0.129)	(0.148)	(0.153)	(0.158)	(0.162)
**Education level (primary school or below)**						
Junior high school	0.376^**^	0.209	0.396^**^	0.249	0.368^*^	0.306
	(0.148)	(0.153)	(0.178)	(0.182)	(0.190)	(0.194)
High school or technical secondary school	0.541^***^	0.206	0.114	−0.183	0.413	0.278
	(0.202)	(0.214)	(0.233)	(0.247)	(0.257)	(0.268)
Junior college or above	1.176^***^	0.779^**^	0.743^*^	0.470	0.930^**^	0.719^*^
	(0.332)	(0.348)	(0.387)	(0.408)	(0.370)	(0.388)
Has spouse (without spouse)	0.271^**^	0.159	0.785^***^	0.734^***^	1.068^***^	0.887^***^
	(0.137)	(0.156)	(0.151)	(0.174)	(0.168)	(0.190)
**Average monthly income (0–1999)**						
2,000–3,999		0.826^***^		0.661^***^		0.267
		(0.163)		(0.194)		(0.200)
4,000 or above		0.759^***^		0.411		0.479
		(0.241)		(0.292)		(0.300)
**Interpersonal expenditure (0–999)**						
1,000–5,999		0.0677		0.457^***^		0.044
		(0.148)		(0.168)		(0.183)
6,000 or above		−0.068		0.261		0.090
		(0.187)		(0.219)		(0.235)
**Living arrangement (alone)**						
With families		0.262		0.186		0.444^*^
		(0.198)		(0.216)		(0.228)
Others		−0.055		0.604		−0.182
		(0.429)		(0.515)		(0.517)
LR *chi^2^*	147.79	179.10	302.60	326.02	78.88	86.88
Pseudo *R*^2^	0.067	0.082	0.176	0.190	0.058	0.063

^*^p < 0.1, ^**^p < 0.05, ^***^p < 0.01.

The reference for each variable is shown in parentheses in column 1. The coefficients are in columns 2–7, where the standard errors are in parentheses.

The results show that smartphone use has a significant positive effect on the self-rated and physical health of older adults at the 1% level, and a significant positive effect on psychological health at the 5% level. Besides, older adults with a higher age are less likely to have better self-rated and physical health. As aging progresses the physical functions of older adults deteriorate, but its impact on psychological health is not significant. In terms of education, all health indicators of older adults have been strengthened with the improvement in education level, which is in line with the findings of Liang et al. (17). Older adults with higher education levels have better physical and psychological health due to their investment in acquiring higher education, and thus receive more rewards, including a better living environment and more knowledge. A spouse has a significantly positive effect on the overall health of older adults, as the companionship of a spouse makes it easier to adapt to changes in their surroundings, thereby reducing the physical and mental shock from changes and improving their health.

Older adults with a higher average monthly income have significantly better self-rated and physical health. The improvement of economic level helps older adults obtain more abundant and convenient health support and better and more timely treatment in case of illness, but the impact of income on psychological health is not significant. Moderate interpersonal expenditure has a significant positive effect on the physical health of older adults. Appropriate social interaction activities not only enhance the social participation of older adults but also increase their daily exercise objectively, thus improving their physical health. Intergenerational support had a significant positive effect on psychological health. Older adults living with their families are more likely to receive emotional support and have better psychological health than those living alone.

### 4.3. Net effects of smartphone use on the health status of older adults

Some studies have shown that older adults who frequently surf the Internet or use smartphones tend to have better health (12, 15, 17). Using smartphones is a choice made by older adults based on their own thoughts, but it may be influenced by objective conditions such as age, gender, and residential area, resulting in the absence of randomized trial premises. The estimated coefficients may have errors owing to self-selection. Based on the reasons above, this study used the propensity score matching model for robustness tests and the net effects, in which three methods, namely, k-nearest neighbor matching, caliber matching, and kernel matching, were used to eliminate the bias caused by mixed factors. As shown in Table 6, the standard deviation of most variables is < 10%, and the differences in most variables between the control and treated groups are not significant after matching. This model largely eliminated self-selection bias, and the sample passed the balance test.

**Table 6:** Sample balance tests.

**Variable**		**Control group**	**Treated group**	**Standard deviation (%)**	**Deviation reduction (%)**	**T-value**	**P-value**
Gender	U	0.411	0.515	21.0	61.0	3.46	0.001
	M	0.550	0.509	8.2			−1.40	0.163
Age	U	2.625	2.010	−93.5		83.5	−15.11	0.000
	M	2.183	2.081	−15.4			−2.53	0.012
Residential area	U	0.361	0.509	30.2	68.0		4.96	0.000
	M	0.538	0.491	−9.7			−1.63	0.103
Education level	U	1.392	1.965	67.6		79.2	10.93	0.000
	M	2.003	1.883	−14.1			−2.09	0.037
Marital status	U	0.589	0.809	49.3	96.2		8.26	0.000
	M	0.787	0.795	1.9			0.35	0.726
Average monthly income	U	1.260	1.727	72.4		93.4	11.65	0.000
	M	1.678	1.651	−4.2			−0.65	0.514
Interpersonal expenditure	U	1.799	2.098	45.3	99.2		7.48	0.000
	M	2.058	2.061	0.4			0.07	0.948
Living arrangement	U	1.235	1.131	−23.8		91.7	−3.99	0.000
	M	1.149	1.140	−2.0			−0.37	0.708

^*^p < 0.1, ^**^p < 0.05, ^***^p < 0.01.

The value in columns 3 and 4 is the mean of the control and treated group, respectively.

According to the results of the propensity score matching analysis (Table 7), the positive contribution of smartphone use to the development of better health among older adults was fully illustrated. The impact of smartphone use on self-rated, physical, and psychological health of older adults obtained from the three matching methods were consistent, and most of them passed the significance test, except the one regarding psychological health. Under control variables, smartphone use led to a significant increase in self-rated health by 0.158–0.178, physical health by 0.118–0.193, and psychological health by 0.063–0.073, compared with older adults who did not use smartphones. This indicates that smartphone use by older adults has a positive effect on all health statuses, and the positive net effects on self-rated and physical health are higher than that of psychological health. At the same time, the significant net effects of the different matching methods are close to each other, which verifies the robustness of the results of the regression analyses.

**Table 7:** Net effect analysis of smartphone use on the health status of older adults.

**Analysis method**		**Control group**	**Treated group**	**Difference**	**SE**	**T-value**
Self-rated health	Unmatched	2.122	2.569	0.447	0.044	10.14
	K-nearest neighbor matching	2.399	2.557	0.158	0.097	1.63
	Caliper matching	2.379	2.557	0.178	0.073	2.45
	Kernel matching	2.379	2.541	0.162	0.072	2.25
Physical health	Unmatched	2.361	2.803	0.442	0.032	13.82
	K-nearest neighbor matching	2.677	2.795	0.118	0.076	1.56
	Caliper matching	2.641	2.795	0.154	0.057	2.72
	Kernel matching	2.594	2.788	0.193	0.056	3.46
Psychological health	Unmatched	1.786	1.919	0.133	0.026	5.04
	K-nearest neighbor matching	1.847	1.910	0.063	0.055	1.14
	Caliper matching	1.898	1.910	0.012	0.043	0.28
	Kernel matching	1.830	1.903	0.073	0.043	1.71

SE, Standard error.

The value in columns 3 and 4 is the mean of the control and treated group, respectively.

### 4.4. Mechanism analyses of the impact of smartphone use on the health status of older adults

As one of the main accesses for older adults to the Internet and interpersonal communication, smartphones play a key role in interactions between older adults and the outside world. In addition to daily needs, such as making phone calls and chatting online by smartphones, older adults also use some applications or search independently to obtain important events or information useful to themselves. Therefore, this study analyzed the mediation mechanism between smartphone use and the health of older adults with mediators, including performance expectations and individualized needs.

As shown in the second column of Tables 8, 9, the effect of smartphone use on performance expectations or individualized needs was significant. The results in Table 5 showed that smartphone use had a significant impact on each health status of older adults. Based on this finding, the mediators were added to the baseline regression equation for each health, and the results were shown in column 3–5 of Tables 8, 9, respectively.

**Table 8:** Mediating role of performance expectations in the relationship between smartphone use and the health status of older adults (*N* = 1,110).

**Variable**	**Performance expectations**	**Self-rated health**	**Physical health**	**Psychological health**
Smartphone use	0.752^***^	0.559^***^	0.913^***^	0.234
	(0.137)	(0.142)	(0.162)	(0.182)
Performance expectations		0.216^***^	0.141	0.269^***^
		(0.082)	(0.094)	(0.101)
Control variable	Yes	Yes	Yes	Yes
Pseudo *R^2^*	0.053	0.085	0.190	0.068
**Test**				
CI		[0.039,0.310]	[-0.032,0.262]	[0.050,0.385]
Indirect effect		0.162	–	0.202

^*^p < 0.1, ^**^p < 0.05, ^***^p < 0.01.

The coefficients are in columns 2–5, where the standard errors are in parentheses.

The control variables included gender, age, residential area, education level, marital status, average monthly income, interpersonal expenditure, living arrangement.

**Table 9:** Mediating role of individualized needs in the relationship between smartphone use and the health status of older adults (*N* = 1,110).

**Variable**	**Individualized needs**	**Self-rated health**	**Physical health**	**Psychological health**
Smartphone use	1.077^***^	0.534^***^	0.896^***^	0.231
	0.138	(0.144)	(0.164)	(0.184)
Individualized needs		0.204^**^	0.137	0.200^*^
		(0.080)	(0.092)	(0.100)
Control variable	Yes	Yes	Yes	Yes
Pseudo *R^2^*	0.078	0.085	0.190	0.066
**Test**				
CI		[0.049, 0.408]	[−0.046, 0.354]	[0.004, 0.445]
Indirect effect		0.220	-	0.215

^*^p < 0.1, ^**^p < 0.05, ^***^p < 0.01.

The coefficients are in columns 2–5, where the standard errors are in parentheses.

The control variables included gender, age, residential area, education level, marital status, average monthly income, interpersonal expenditure, living arrangement.

The results showed that the performance expectation had a significant effect on the self-rated health and psychological health of older adults when it was used as a mediator, but did not show a significant effect on physical health. Further, the impact of smartphone use on self-rated health decreased, and the impact on psychological health was no longer significant after adding this mediator, indicating that the performance expectation played a major mediating role between smartphone use and self-rated health, and the major mediating role between smartphone use and psychological health was even more pronounced than the direct effect between them. Next, the distribution-of-product method was used to test the existence of the mediation effect. The results of the test were consistent with the previous results, the mediating effect of the performance expectation between smartphone use and self-rated health was 0.162, between smartphone use and psychological health was 0.202.

When the individualized need was used as the mediator, the results were similar to that of the performance expectation. The individualized need also played a major mediating role between smartphone use and self-rated health, and between smartphone use and psychological health, which all passed the test of distribution-of-product method. The mediating effect of the individualized need between smartphone use and self-rated health was 0.220, between smartphone use and psychological health was 0.215.

The analysis results showed that the higher the satisfaction of older adults' individualized needs, the better their health status. On the one hand, older adults will not suffer from the psychological burden of embracing life, so they can spend extra time contacting more advanced intelligent technology, broaden their horizons, and make themselves happy. Thus, they can improve their self-regulatory abilities and health levels. On the other hand, they can maintain an active state, and be socially involved based on a more prosperous life, and better alleviate their sense of loneliness. Second, older adults can enhance their health literacy by acquiring valuable, useful information. They can improve their adaptability to the outside world and overall health, while enriching their inner thoughts by acquiring valuable information regarding enhancement of their cognitive realm.

### 4.5. Heterogeneity analyses of the impact of smartphone use on the health status of older adults of different age and residence

These results indicate that smartphone use can improve the overall health of older adults, but the impact of smartphone use on their health status may vary depending on the characteristics of older adults. The heterogeneity analysis of each health status of older adults demonstrated differences in the impact of use among different groups of older adults (Tables 10, 11).

**Table 10:** Impact of smartphone use on the health status of older adults at different ages.

**Variable**	**Self-rated health**			**Physical health**			**Psychological health**		
	**55–64**	**65–74**	**75 or above**	**55–64**	**65–74**	**75 or above**	**55–64**	**65–74**	**75 or above**
Smartphone use	0.754 (0.550)	0.241 (0.218)	0.948^***^ (0.198)	1.851^**^ (0.783)	0.630^**^ (0.260)	1.116^***^ (0.210)	−0.248 (0.736)	0.133 (0.278)	0.558^**^ (0.254)
Control variable	Yes	Yes	Yes	Yes	Yes	Yes	Yes	Yes	Yes
Observations	191	424	495	191	424	495	191	424	495
Pseudo *R^2^*	0.032	0.045	0.072	0.135	0.074	0.091	0.061	0.083	0.057
**Empirical** ***p-*****value**									
55–64 vs. 65-74	0.122			0.098^*^			0.285		
55–64 vs. 75 or above	0.302			0.085^*^			0.050^*^		
65–74 vs. 75 or above	0.001^**^			0.055^*^			0.115		

^*^p < 0.1, ^**^p < 0.05, ^***^p < 0.01.

The coefficients are in columns 2–10, where the standard errors are in parentheses.

The empirical *p*-value obtained from bootstrap sampling (1,000 times) was used to test the significance of the between-group difference in coefficients.

The control variables included gender, age, residential area, education level, marital status, average monthly income, interpersonal expenditure, living arrangement.

**Table 11:** Impact of smartphone use on the health status of older adults with different residences.

**Variable**	**Self-rated health**		**Physical health**		**Psychological health**	
	**Urban**	**Rural**	**Urban**	**Rural**	**Urban**	**Rural**
Smartphone use	0.826^***^ (0.210)	0.392^**^ (0.188)	1.111^***^ (0.229)	0.750^***^ (0.223)	0.398 (0.263)	0.182 (0.247)
Control variable	Yes	Yes	Yes	Yes	Yes	Yes
Observations	494	616	494	616	494	616
Pseudo *R^2^*	0.061	0.090	0.148	0.226	0.058	0.075
Empirical *p*-value	0.053^*^		0.128		0.294	

^*^p < 0.1, ^**^p < 0.05, ^***^p < 0.01.

The coefficients are in columns 2–7, where the standard errors are in parentheses.

The empirical *p*-value obtained from bootstrap sampling (1,000 times) was used to test the significance of the between-group difference in coefficients.

The control variables included gender, age, residential area, education level, marital status, average monthly income, interpersonal expenditure, living arrangement.

As can be seen from the age subsample regression results, first, smartphone use had a significant positive effect on the physical health of older adults of all ages. And there were significant differences in the effects of older adults among all age groups. The smartphone use showed a positive effect on self-rated and psychological health only of the older adults aged 75 or above. Second, in terms of the size of the regression coefficients, the impact of smartphone use on older people of different ages did not change regularly with age in addition to psychological health; that is, active older adults aged 65–74 years are relatively less affected, followed by the older adults aged 55–64, older adults aged 75 or above are the most positively affected. Regarding psychological health, the impact grows with age, and older adults aged 55–64 years are negatively affected. Older adults of younger age are more likely to be more receptive to smart devices such as smartphones and less likely to have difficulty using them, as they are more similar to the smart age in their middle and younger years. Older adults with a higher age have relatively less access to information in modern society, and the marginal impact of outside information on them through smartphones can be greater than that of other channels. It is important to note that older adults aged 55–64 should be aware of digital addiction due to overuse and prevent the consequent negative psychological health effects (30).

In terms of the regression results for the residential subsample, first, smartphone use showed a significant effect on self-rated and physical health in both urban and rural older adults but not on their psychological health. It is notable that there is a significant difference in the effect on self-rated health between urban and rural older adults. Second, the size of the regression coefficients in urban older adults was larger than that in rural older adults for each health status. From the results of the previous analyses, urban older adults have a higher proportion of smartphone use and more access to new technologies such as smart devices. Urban older adults are more fluent and less psychologically frustrated when using smartphones and are better able to obtain benefits and convenience from the process based on the advanced development of urban areas. Further digitalization in rural areas is needed to promote the digital literacy of rural older adults, which in turn improves their health literacy.

Above all, according to the findings we can see that, smartphones do have a significant impact on the health status of older adults on the whole. From the regression results and propensity score matching results we know that, smartphones have a significant positive impact on self-rated health, physical health and psychological health of older adults. At the same time, different age, residences, education level, marital status and other characteristics also have an obvious impact on the health status of older adults. For older adults of different age and residences, the impact of smartphone use on health differs significantly. As for the impact of other factors, the individualized needs of older adults play a significant mediating effect in the impact of smartphone use on various health, and performance expectations play a significant mediating effect in the impact of smartphone use on self-rated health and psychological health.

## 5. Discussion

The above findings suggest that smartphones have an enhancing impact on the self-rated, physical, and psychological health of older adults, especially on their physical health. At the same time, older adults' performance expectations play a significant partial mediating role between smartphone use and self-rated and psychological health. This finding further deepens the conclusion obtained from existing studies that health training through smartphones can improve the health of older adults (16). Further, we also found that older adults' individualized needs have a significant partial mediating effect between smartphone use and each health status. Older adults in this era are more focused on the usefulness and tangible value of objects, so they may be more concerned about what smart devices, such as smartphones, can bring to life. Therefore, access to knowledgeable information and satisfaction with life needs are the best outcomes that older adults expect to obtain through worthwhile economic exchange. Use of smartphones is convenient while also playing a key role in the health status of older adults.

However, this study found that smartphone use has a greater effect on the health of urban older adults compared to rural older adults, contrary to the findings of regional heterogeneity in Internet use (20). On the one hand, the marginal effect of smartphones and internet on rural older adults today is slightly weaker than it used to be, with the gradual optimization of rural construction. On the other hand, urban areas still have superior environments and living conditions compared to rural areas, where urban older adults are more likely to obtain the actual effect of digital support services in real life. The actual digital impact that rural older adults can receive is relatively weak, as digital construction in rural areas still needs to be improved. In terms of controlling factors, the effect of social engagement found by Zhang and Li (13) was limited in this study. A possible reason for this is that the interpersonal expenditure investigated in this study is the total spending of the household rather than that of the individual older person. It is influenced by the social interaction of their offspring, and therefore cannot accurately reflect the social support of the older person separately.

The above findings reflect the importance of smart device popularization in promoting the health of older adults and the construction of a healthy China. The digital integration dilemma of older Chinese people is becoming increasingly prominent with the development of the smart age. China needs to continuously promote the digital integration of older adults to enhance their overall life, including their health status. In recent years, China has introduced several policy documents to address the difficulties of older adults in accessing and using smart technologies and to expand the use scenarios in which smart devices can meet the needs of older adults. Based on the findings of this study, we should continue to promote access and smart device use among older adults. First, we should provide public service guidance for older adults to improve their digital skills, increase publicity in smart device use for older adults, and improve their awareness of the usefulness of smart devices in daily life and social interactions. Second, investment in the construction of digital infrastructure in rural areas should be expanded to improve the access and use of smart devices for rural older adults. Third, age-oriented requirements should be incorporated into the formulation of technical standards for smart devices to enhance the willingness of older adults to use them with the convenience of operation. Fourth, collaborative research between digital health professionals and typical older adults needs to be promoted to conduct in-depth study on the impact of smart devices on older adults' health. Thus, we can help improve older adults' digital literacy and help them integrate into the necessary digital life under the normalization of epidemic prevention.

## 6. Limitations

This study does have some limitations. First, the study takes smartphones as the representative of smart devices, which is rather representative but still not enough, and more studies on smart device use, such as smart watches or smart homes, can be subsequently added. Second, the data in this study were cross-sectional rather than long-term. It is less possible to obtain comparative differences before and after using the same sample; therefore, causal inferences cannot be made. Third, the level is not detailed enough when sampling, resulting in the uneven distribution of urban and rural areas of the respondents, which may make it difficult to detect the difference in impact between urban and rural older adults Further research can track changes in the same group of older adults before and after the use of smart devices to enrich the relevant research results. Combined with the findings and the limitations of the survey scope, the scope of the survey can be expanded to the whole country which can based on the existing nationwide survey, so that the impact of smart device use in China can be analyzed in more ways.

## 7. Conclusion

This paper reports the findings of a quantitative analysis to investigate the association between smart device use and health status of older adults in China. This analysis is the first study combined three dimensions of health to examine the effect of smart device use on health status and takes the real-life effect into account. The results of regression analysis showed that smartphone use could play an important role in improving the self-rated, physical and mental health of older adults, and subsequent propensity matching analysis also verified this finding. The age, education level, marital status, economic level and intergenerational support of older adults could significantly affect the physical health of older adults, among which the education level, marital status and economic level played an important role in the overall health of older adults. The results of the mediation effect test showed that the influence of smartphones on the health of older adults in real life would be affected by the perceived practicality of older adults, which also reflected the multi-faceted practical impact of the application effect in real scenarios. The results of heterogeneity analysis showed that, compared with older adults with a young age, older adults with a higher age were more likely to be affected by the positive effect of smartphones, while the former may experience digital addiction, which also provided a further direction for the improvement of smartphones for aging. As for people in different residence, the positive impact is stronger among urban older adults, with a more pronounced gap between urban and rural older adults in terms of self-rated health.

## Data availability statement

The raw data supporting the conclusions of this article will be made available by the authors, without undue reservation.

## Author contributions

YW developed the main ideas of the study, gathered the data, and proofread the article. XG performed the model construction and estimation, wrote and edited the manuscript. Both authors have read and agreed to the published version of the manuscript.
